# Comparison of Static and Microfluidic Protease Assays Using Modified Bioluminescence Resonance Energy Transfer Chemistry

**DOI:** 10.1371/journal.pone.0088399

**Published:** 2014-02-14

**Authors:** Nan Wu, Helen Dacres, Alisha Anderson, Stephen C. Trowell, Yonggang Zhu

**Affiliations:** 1 CSIRO Materials Science and Engineering and Food Futures Flagship, Clayton South, Australia; 2 CSIRO Ecosystem Sciences and Food Futures Flagship, Canberra, Australia; CNR, Italy

## Abstract

**Background:**

Fluorescence and bioluminescence resonance energy transfer (F/BRET) are two forms of Förster resonance energy transfer, which can be used for optical transduction of biosensors. BRET has several advantages over fluorescence-based technologies because it does not require an external light source. There would be benefits in combining BRET transduction with microfluidics but the low luminance of BRET has made this challenging until now.

**Methodology:**

We used a thrombin bioprobe based on a form of BRET (BRET^H^), which uses the BRET^1^ substrate, native coelenterazine, with the typical BRET^2^ donor and acceptor proteins linked by a thrombin target peptide. The microfluidic assay was carried out in a Y-shaped microfluidic network. The dependence of the BRET^H^ ratio on the measurement location, flow rate and bioprobe concentration was quantified. Results were compared with the same bioprobe in a static microwell plate assay.

**Principal Findings:**

The BRET^H^ thrombin bioprobe has a lower limit of detection (LOD) than previously reported for the equivalent BRET^1^–based version but it is substantially brighter than the BRET^2^ version. The normalised BRET^H^ ratio of the bioprobe changed 32% following complete cleavage by thrombin and 31% in the microfluidic format. The LOD for thrombin in the microfluidic format was 27 pM, compared with an LOD of 310 pM, using the same bioprobe in a static microwell assay, and two orders of magnitude lower than reported for other microfluidic chip-based protease assays.

**Conclusions:**

These data demonstrate that BRET based microfluidic assays are feasible and that BRET^H^ provides a useful test bed for optimising BRET-based microfluidics. This approach may be convenient for a wide range of applications requiring sensitive detection and/or quantification of chemical or biological analytes.

## Introduction

Biosensors promise rapid, sensitive and selective estimation of a wide range of analytes [1]–[3]. Bioluminescence resonance energy transfer (BRET) is a form of Förster resonance energy transfer, the non-radiative transfer of energy from an excited state donor to a ground state acceptor, which can be used to transduce biosensor activation into a machine-readable format. Compared to amplitude-based measurements, the ratiometric nature of Förster resonance energy transfer reduces signal variability from a variety of sources including variations in assay volume, minor temperature variations and time dependent signal decay. RET-based reactions are homogeneous and can be performed in the fluid phase without solid-phase attachment. This allows for detection of analytes in the fluid phase or particulate suspensions in fluids, without the need for separation. This has many advantages, including the ability to make continuous measurements in a flow format, without having to regenerate a sensing surface, as required, for example by surface plasmon resonance based biosensors [4].

BRET occurs naturally in marine organisms such as *Aequorea victoria* and *Renilla reniformis*. Unlike fluorescence resonance energy transfer (FRET), BRET does not involve an external light source, and is therefore largely free from autofluorescence, light scattering, photobleaching and/or photoisomerization of the donor moiety [5], all of which can limit the sensitivity of FRET. Absence of these effects results in a low background and very low limits of detection for BRET assays [6]. Due to these characteristics, BRET has been used for a variety of applications including RNA detection [7], investigating protein-protein interactions [8]–[10], drug screening [5], [9], [11], imaging [8], [12], [13], and general biosensing [14], [15]. So far, however, uses of BRET have largely been restricted to fundamental research, often using sophisticated imaging equipment.

Despite its lower sensitivity, FRET has been used more broadly than BRET for screening and biosensing applications. Recently, however, we demonstrated that a form of BRET is 50 times more sensitive than FRET for measuring thrombin-catalysed proteolytic cleavage of a target peptide sequence in a microplate assay [16]. A microfluidic format offer several advantages over static microplate assays but the generally low luminance of some BRET systems makes it quite challenging to detect a signal in the small volumes typical of a microfluidic device. Therefore, although substantial effort has been invested in developing FRET detection systems and bioluminescence detection for microfluidic systems [17]–[22], there have been very few studies combining BRET-based sensors with microfluidics.

The aim of this study was therefore to test the feasibility, sensitivity and limits of detection of a homogenous BRET-based biosensor in a microfluidic format, compared with the same biosensor in a conventional assay. We selected a thrombin bioprobe [6], [23] for this work because it is well characterised, highly specific and clinically relevant.

In earlier work we used the BRET^2^ system because of its long Förster distance [24], sensitivity and its potential application to measuring intramolecular rearrangements [6], [25] as well as dissociations. Unfortunately, however, the BRET^2^ system was not bright enough to be detected in the microfluidic system used here, due to its well-documented low quantum yield (e.g., Pfleger *et al.* [9], [26]) and the small volume (2.6 nL) sampled optically. For comparison, the standard volume optically sampled in the 96 well microplate format is 100 µL. We therefore adopted a novel BRET variant, which we named BRET^H^, combining the donor and acceptor domains of BRET^2^: i.e. GFP^2^ and RLuc, with the original BRET^1^ substrate, native coelenterazine. This provides much greater luminosity with limits of detection intermediate between BRET^1^ and BRET^2^, at the expense of an undefined, presumably shorter, Förster distance. For an assay involving complete molecular dissociation, such as the one here, a short Forster distance is of lesser concern. This compromise allowed us to compare the static and microfluidic versions fo the assay without the inconvenience of having to substitute the BRET^2^ donor and acceptor proteins. To the best of our knowledge, this is the first realisation of a BRET based biosensor in the fluid phase of any microfluidic system. The work lays the foundation for a new class of BRET based sensing devices for a wide range of analytes.

The limit of detection for thrombin in the microfluidic format was 27 pM, which was more than tenfold lower than when measured using the same sensor in a microwell plate and two log units lower than comparable FRET-based microfluidic assays. The sensitivity of our microfluidic method was approximately five times greater than when using a microwell plate. This result demonstrates the feasibility of developing ultra-sensitive, continuous flow miniature BRET-based biosensing devices and provides a convenient testbed for optimising overall system design.

## Methodology and Experimental Details

### BRET System

**Figure 1:**
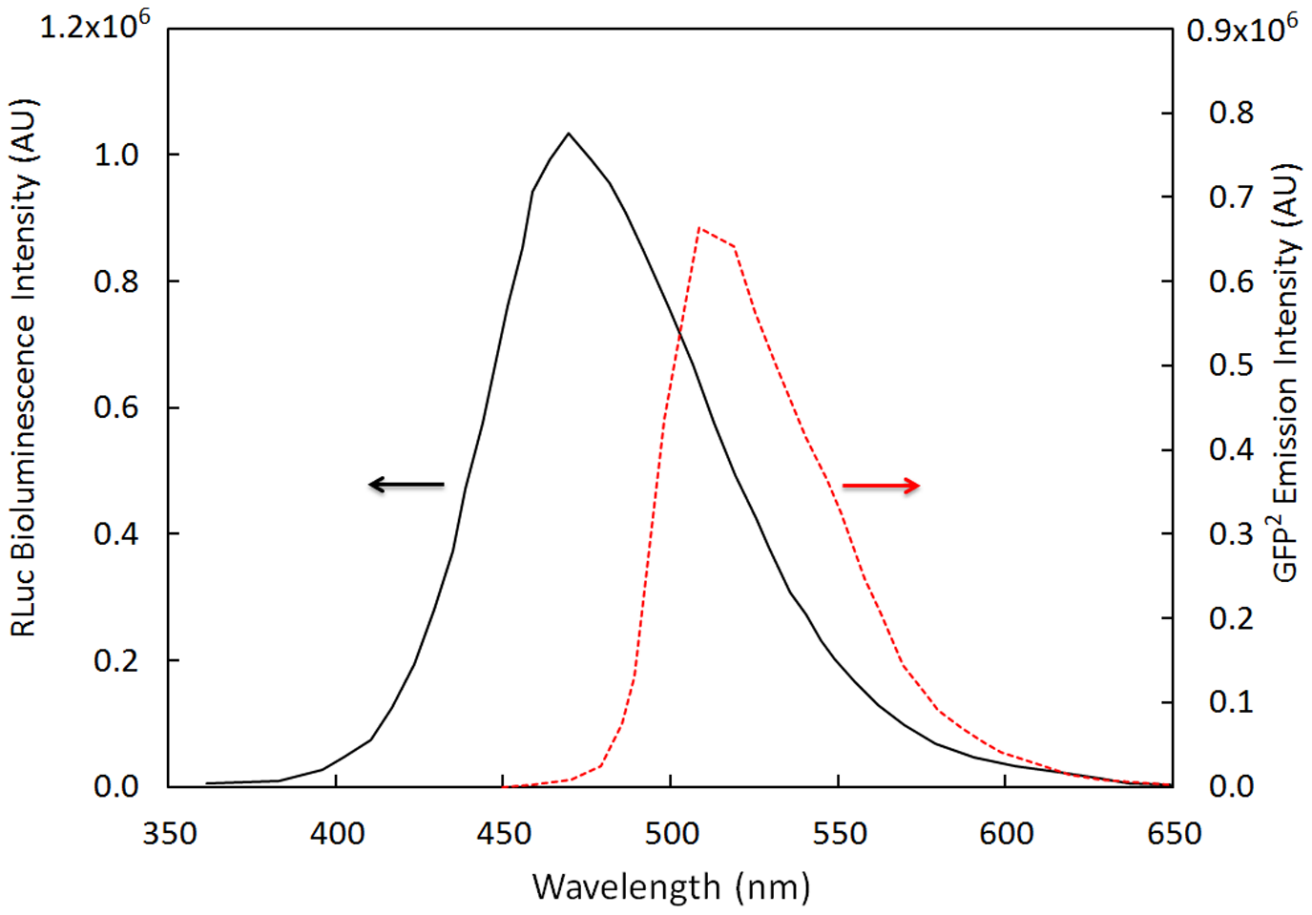
Emission spectra of RLuc and GFP^2^. Rluc emission spectrum were generated using 1^2^ emission spectrum were generated using λ_ex_ of 420 nm and 1 uM GFP^2^ protein.

**Figure 2:**
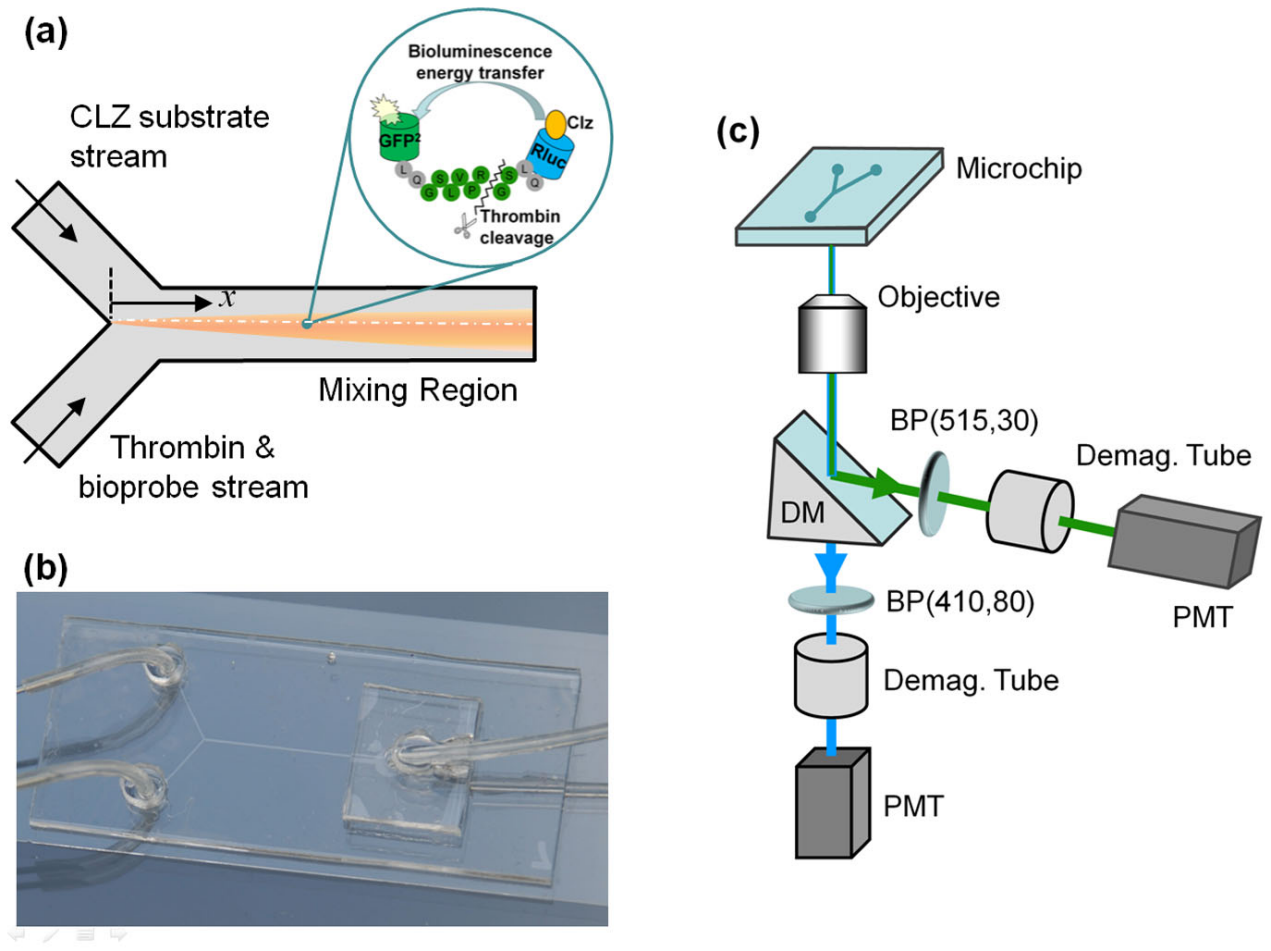
Experimental set-up. (a) The arrangement of the microchannel network with the origin for distance measurements and a schematic drawing of the BRET^H^ reaction. (b) A picture of the microfluidic device. (c) Schematic of the optoelectronic detection apparatus. (Clz = Native coelenterazine, DM = dichroic mirror, BP = band pass, PMT = photomultiplier tube).

Aspects of the BRET^1^ and BRET^2^ systems were combined into a hybrid version of BRET, we named BRET^H^. Specifically, RLuc with native coelenterazine substrate was used as the bioluminescent donor and GFP^2^ as the acceptor molecule. The emission spectra of RLuc and GFP^2^ have peaks at 470 nm and 500 nm, respectively, as shown in Figure 1. The donor and acceptor were linked by a linker peptide containing the thrombin cleavage sequence: LQGSLVPR↓GSLQ (RG). Cleavage of the linker results in a change in the hybrid BRET^H^ signal. The assay principle is shown in Figure 2(a).

### Materials

The GFP^2^-RG-RLuc bioprobe was expressed in *E. coli* and purified as reported previously [6]. The purified fusion protein was re-suspended in thrombin cleavage buffer (10 mM Tris (pH 8.0), 100 mM NaCl, 1 mM EDTA). The final concentrations of the native coelenterazine substrate (Biosynth) were 58.6 µM for microfluidic based assays and 5 µM for plate-reader based assays. 1 unit (U)/µl thrombin protease (Amersham Biosciences) solution was prepared in phosphate buffered saline (PBS).

### Experimental Set-up

Simultaneous dual emission hybrid BRET measurements were carried out in a microplate using a SpectraMax M2 spectrofluorometer (Molecular Devices) in luminescence scan mode between wavelengths of 400 to 650 nm and in the microfluidics apparatus described below.

For the microchip experiment, a simple Y-shape microchannel (Figure 2(b)), 70 µm wide and 50 µm high was used which was fabricated in polydimethylsiloxane (PDMS) using standard photolithography. The chip design was completed in a commercial drawing package (Adobe Illustrator CS4) and the design pattern was printed on a transparency mask (5,080 dpi, Allardice). Master patterns of the microfluidic devices were fabricated using a laminar dry film resist (Shipley 5038). Multiple layers of resist were laminated at 113°C onto a substrate of polished stainless steel. The channels were lithographically patterned using a collimated UV source (λ = 350–450 nm) operated at 20 mJ/cm^2^ and a transparency film mask. After exposure, the test pattern was developed in a 20% Na_2_CO_3_ solution. The pattern in resist was subsequently replicated as a Nickel shim using an initial sputter deposition of 100 nm Ni followed by electroplating to a thickness of 150 µm. A 10/1 (w/w) ratio of PDMS and curing agent was poured over the shim, degassed and baked overnight at 75°C. The device was cut and peeled off the shim and exposed to air plasma for 10 minutes. The PDMS was immediately sealed with a glass slide. After baking for three hours at 75°C, the PDMS adhered strongly to the surface of the glass and the PDMS glass microchip was ready to use.

A schematic of the set-up for microfluidic measurement is shown in Figure 2(c). A neMESYS high pressure pump system (Cetoni, Germany) was used to pump the two fluids from two 50 µL SGE syringes (Supelco) onto the microchip, respectively. One fluid is the substrate solution while the other is a mixture of thrombin and bioprobe solutions. The flow rates of both streams were 20 µl/h. The microchip was placed on a microscope (Nikon Eclipse TE2000-U) stage for visualization and measurement. A sapphire laser (488 nm, Coherent) was used to locate the detection spot for each experiment. The bioluminescence emissions from the detection spot of the microchannel were collected with a 20× objective lens (Plan Fluor, Nikon). A dichroic mirror was used to split the light into two separate channels with a splitting wavelength of 505 nm. Bandpass filters (Nikon) of 515–555 nm for GFP^2^ and 430–455 nm for RLuc were used for the two channels. De-magnification lenses (Nikon C-0.45×) were used to focus light emitted from each channel onto separate photomultiplier tubes (Hamamatsu H7421). Integration time for data acquisition was 200 ms for both photomultipliers. The measurement position was varied along the main channel starting at the confluence of the input channels (x = 0) to the end of the common channel (x≈6.9 mm).

### Thrombin Assay

Various concentrations of thrombin were added to the purified GFP^2^ -RG-RLuc bioprobe and incubated at 30°C for 90 minutes. To measure the extent of thrombin cleavage following incubation, the reaction mixture and the native CLZ solution were pumped from separate syringes through the two inlet channels and into the common channel. Diffusion of the substrate and the bioprobe energised a BRET reaction that started at the interface and spread into the two streams. As a negative control, recombinant hirudin (Sigma), which is known to stably inhibit thrombin by binding to its catalytic and other sites [27], was incubated with thrombin at room temperature for ten minutes prior to the protease assay.

### Sodium Dodecyl Sulfate Polyacrylamide Gel Electrophoresis (SDS-PAGE)

Proteins (2.5 µg) were diluted in 1× sample-loading buffer for SDS-polyacrylamide gel electrophoresis on a 12% Bis-Tris gel with MOPS running buffer (NuPAGE Invitrogen, Australia). After electrophoresis, the gels were washed, three times ten minutes, in 60 ml of 45% (v/v) methanol, 10% (v/v) acetic acid at room temperature with gentle agitation. The gel was submerged overnight in 16% (v/v) Fast StainTM (Fisher Scientific, Australia) with gentle shaking. Gels were de-stained by washing three times for ten minutes in 10% (v/v) acetic acid with gentle shaking. Gels were photographed with transmitted fluorescent illumination using an AlphaImager™ 2200 video capture system (Alpha Innotech Corporation).

### Data Analysis

Using the microplate spectrophotometer, BRET^H^ ratios were calculated as the ratio of the luminance intensity at λ = 500 nm and λ = 470 nm. For the microfluidic chip, the hybrid BRET^H^ ratio was calculated as the ratio of the luminances in the longer wavelength band (λ = 515–555 nm) and the shorter wavelength band (λ = 430–455 nm) [26]. To allow comparison between the two different detection systems, BRET^H^ ratios were normalized by expressing them as a multiple of the BRET ratio without added thrombin, measured in the same system. Limits of detection (LOD) were formally calculated as the concentration of analyte required to give a signal equal to the background (blank) plus three times the standard deviation of the blank. All data are reported as means ± standard deviation (SD). Two-tailed unpaired t-tests were performed using Graphpad prism (version 5.00 for Windows, Graphpad Software, San Diego, California, USA). Statistical significance is defined as p<0.05.

## Results and Discussion

### Microplate Measurements of BRET Spectra and Ratios

**Figure 3:**
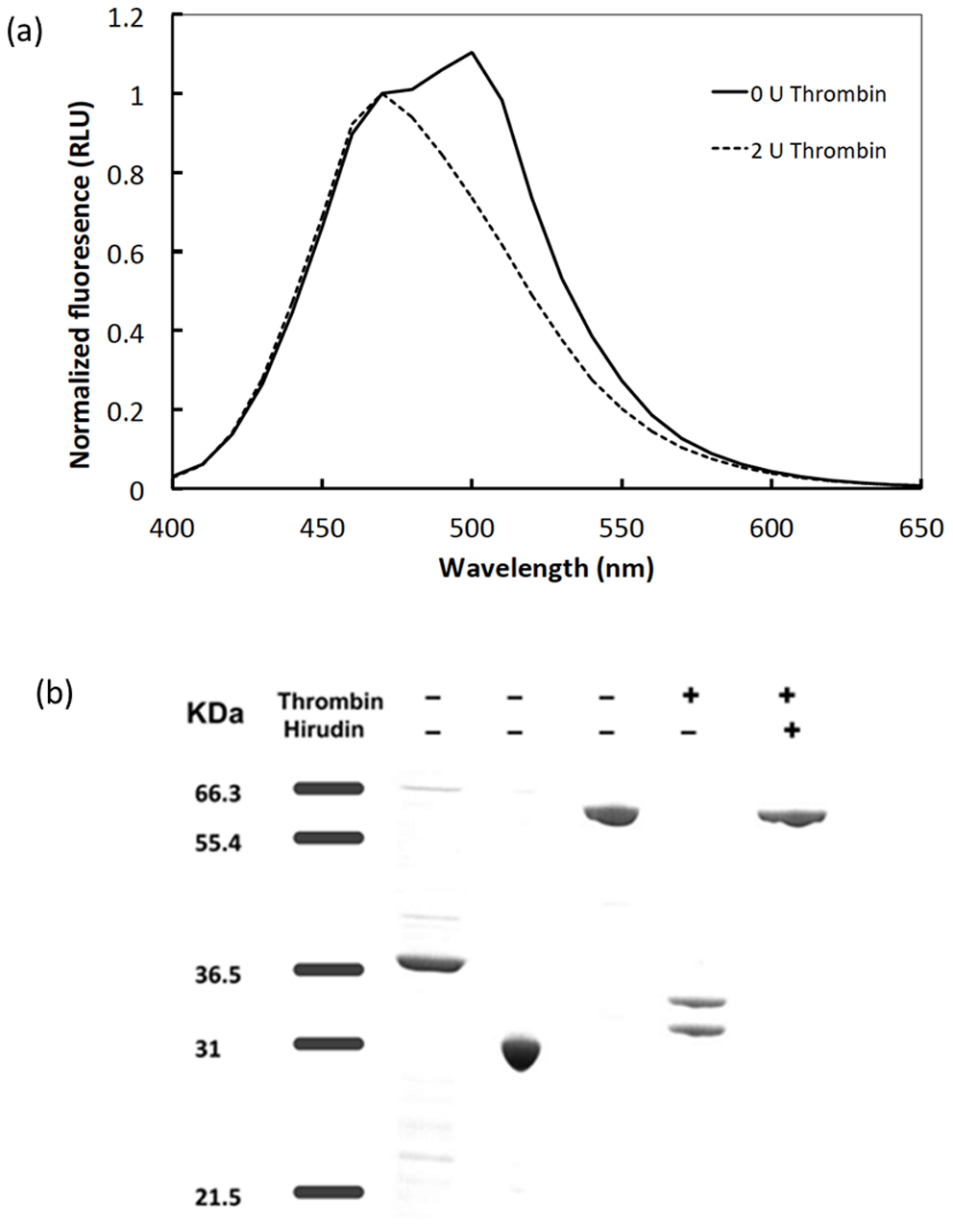
Thrombin cleavage of the bioprobe. (a) Normalised luminescence spectra of GFP^2^-RG-RLuc +5 µM native coelenterazine with or without prior incubation with 2 units of thrombin. Spectra were normalised by dividing by the Rluc peak intensity at 470 nm. (b) SDS-PAGE analysis of cleaved and intact bioprobe and purified His-tagged BRET proteins. 2.5 µg protein loaded per lane. Lane 1: Molecular markers (KDa), Lane 2: RLuc, Lane 3: GFP^2^, Lane 4: intact GFP^2^-RG-RLuc, Lane 5: GFP^2^-RG-RLuc following incubation with 54 nM thrombin for 90 minutes at 30°C, Lane 6: GFP^2^-RG-RLuc, pre-incubated with 2 units of hirudin for 10 minutes at room temperature prior to thrombin treatment as for Lane 5.

The bioluminescence spectrum of the bioprobe in the absence of thrombin was bimodal (Figure 3a), with a peak at 470 nm representing RLuc emission and a second peak at 500 nm representing GFP^2^ emission, indicating efficient energy transfer from the excited state of native coelenterazine to GFP^2^. Following incubation with 2 units of thrombin for 90 minutes, the 500 nm peak was abolished, indicating reduced efficiency of energy transfer from donor to acceptor due to complete cleavage of the thrombin bioprobe. It should be noted that the temperature optimum for Rluc/coelenterazine (e.g. BRET^H^ donor) is 32°C and the pH optimum is pH 7.4. The enzyme will withstand incubation at temperature up to 45°C for 1 h without loss of activity and it is stable within a pH range of 6 to 10 [28].

SDS-PAGE (Figure 3b) showed that, following thrombin treatment, the fusion protein was cleaved into two components with molecular weights of 32.4 KDa and 36.4 KDa (Lane 5, Figure 3b) corresponding to His-tagged GFP^2^ and untagged RLuc. Pre-incubation of the BRET bioprobe with hirudin, a potent thrombin inhibitor, inhibited the cleavage confirming the thrombin dependency of the reaction (Lane 6, Figure 3b).

**Figure 4:**
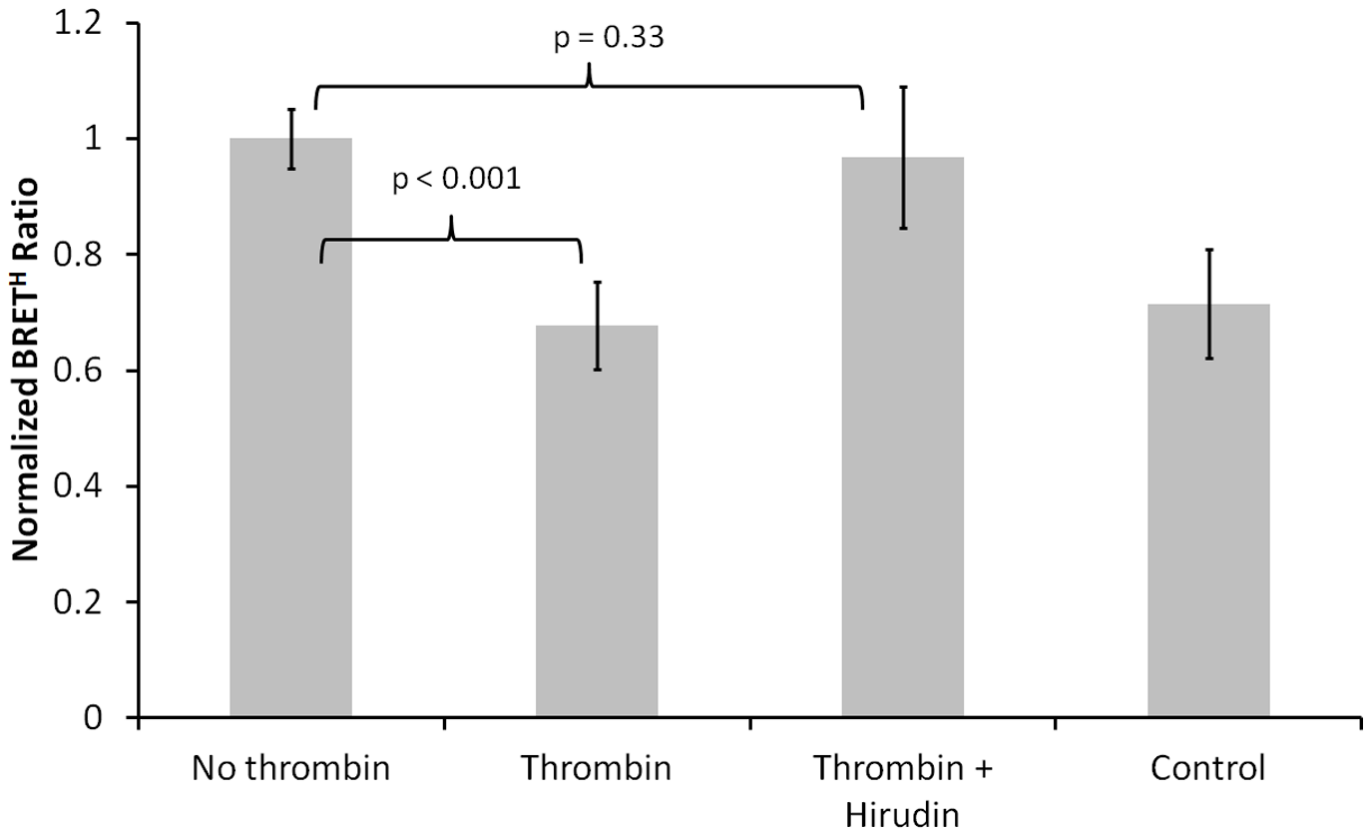
Dependence of bioprobe BRET^H^ ratio (500 nm/470 nm) on thrombin pre-treatment (mean ± S.D., n = 3). All treatments included 1 µM GFP^2^-RG-RLuc. The control was 1 µM RLuc, 1 µM GFP^2^ and 5 µM native coelenterazine. Thrombin pre-treatment was 54 nM thrombin for 90 minutes at 30°C. The thrombin+Hirudin condition involved addition of 2 units of hirudin at room temperature 10 minutes before addition of 54 nM thrombin. All data were measured in a 96 well microplate using a SpectraMax M2 spectrofluorometer.

The change in BRET^H^ ratio due to bioprobe cleavage by thrombin (Figure 4) was approximately 32%, from 1.1±0.06 to 0.75±0.04 (P = 0.001). The BRET^H^ ratio following thrombin cleavage was not significantly different (P = 0.33) from the BRET^H^ ratio (0.79±0.05) of a mixture of 1 µM each of RLuc and GFP^2^. Pre-addition of hirudin stabilised the BRET^H^ ratio at 1.1±0.14, not significantly different (P = 0.77) from the BRET^H^ ratio of the untreated bioprobe. These results show that bioprobe cleavage depends absolutely on and can be used to detect thrombin activity. We have previously shown that the thrombin site in this bioprobe is resistant to caspase, an unrelated protease [16].

### Measurement of BRET^H^ on Chip

**Figure 5:**
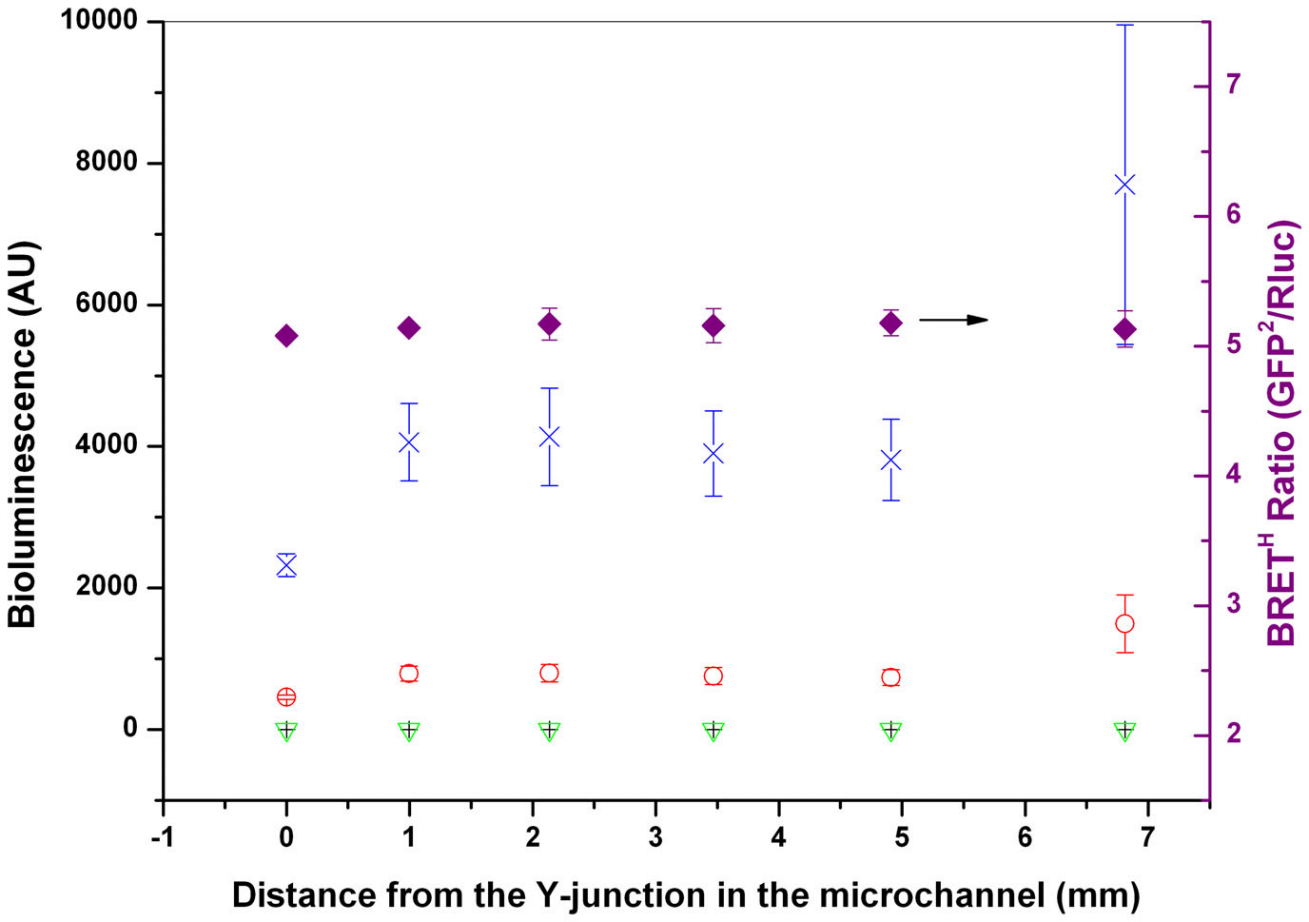
Variation in luminescence with distance along the common microfluidic channel. GFP^2^-RG-RLuc luminescence intensities (AU) for RLuc (+) and GFP^2^ (▿) channels in the absence of and for RLuc (○) and GFP^2^ (×) channels in the presence of coelenterazine substrate. (♦) BRET^H^ ratio as a function of distance x from the Y-junction as labelled in Figure 6(b). The concentration of GFP^2^-RG-RLuc was 3.0 µM and the concentration of coelenterazine was 58.6 µM. [The higher substrate concentrations used in microchip is to increase the light output from the much smaller volume.] Flow rate in the common channel was 40 µl/h. Other conditions as described in “Experimental” and Figure 6.

Thrombin activity was measured in a microfluidic device (Figure 1). To optimize flow conditions, experiments were carried out to image and quantify the BRET^H^ luminance at different positions, flow rates and bioprobe concentrations (Figure 5). In the initial stage of contact between the two fluid streams, cross diffusion of bioprobe and substrate molecules into opposite streams is just beginning. Therefore only a small region of the fluids near the centreline emits light (data not shown). As *x* increases along the channel, the lateral cross-diffusion of the bioprobe and substrate increases as does the width of the bioluminescent band. However, due to the large difference in molecular weights of the substrate (423.4 Da) and bioprobe (∼67 kDa), the diffusion rates of the two molecules are very different, resulting in an asymmetric bioluminescent band along the centreline. Assuming the diffusivity coefficients are ∼10^−10^ m^2^/s and ∼10^−11^ m^2^/s for the substrate and bioprobe, respectively, the width of the substrate diffusional band is calculated to be 3–4 times that of the bioprobe diffusional band (see Figure S1 in the electronic supplementary information). Dark field photography (not shown) of the junctional region of the microchannel confirmed this estimation. Because concentrations of the substrate and bioprobe are highest along the centreline all subsequent measurements were carried out along the midline of the main channel. We found that µM concentrations of coelenterazine, i.e ≈1,000 fold higher than optimal in the multiwell plate, gave the best luminance in the microfluidic format, presumably because of enhanced substrate diffusion into the bioprobe stream. Because of the low volumes used, this was cost effective. The initial intensity of the bioluminescence was low, albeit significantly higher than the background, with approximately 460 arbitrary light units (AU) in the RLuc channel and 2,100 in the GFP^2^ channel, compared with a background ≈2 AU. For locations from x = 1 to 5 mm, the bioluminescence intensities were higher (≈800 and 4,000 AU) and virtually constant but there was a significant increase at x = 6.9 mm, which may reflect more complete mixing in this region. Regardless of the intensity of the bioluminescence, the BRET^H^ ratio remained almost constant (≈5.2) throughout the entire measurement region. The large numerical difference between the BRET^H^ ratios measured in the multiwell plate and the microfluidic chip are attributed to the necessarily different detection equipment used. To benchmark on-chip measurements, those BRET ratios were compared with measurements made using a SpectraMax M2 spectrofluorometer (Molecular Devices). The relative changes in BRET^H^ ratio, measured with and without thrombin, were the same within ±4% for both microchip and microplate systems.

**Figure 6:**
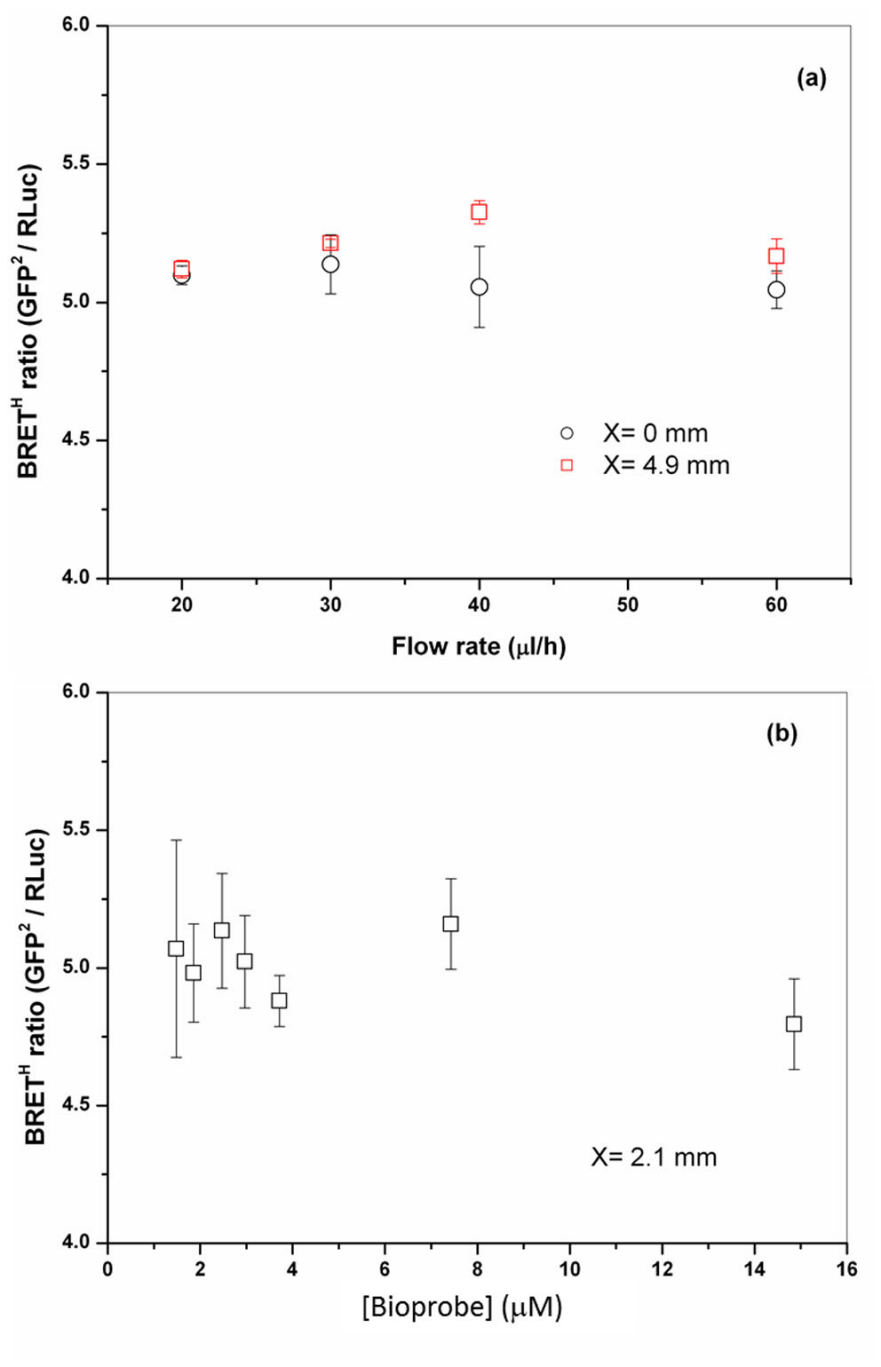
BRET^H^ ratios as a function of total flow rate of the aqueous streams (a) and the bioprobe concentration (b) (mean ±SD, n = 5). (a) Fusion protein concentration was 3.0 µM and native coelenterazine concentration was 58.6 µM; (b) Native coelenterazine concentration was 58.6 µM; each aqueous flow rate was 20 µl/h; a 20× objective was used; filter band pass for GFP^2^ and RLuc channel are 515–555 nm and 430–455 nm respectively; an internal gate time of 200 ms was used for data acquisition.

The flow rate dependency of the BRET^H^ ratio was measured at two locations, *x* = 0 and 4.9 mm (Figure 6a). At *x* = 0, the BRET^H^ ratio was constant (±1.1%) over the full range of flow rates studied, i.e. 20–60 µl/h. At *x* = 4.9 mm, there was also very little variation (±2%) in the BRET ratio with changing flow rate. The overall uncertainty in BRET^H^ ratios between the two measurement locations was within ±5%. The mean BRET^H^ ratio did not vary systematically with bioprobe concentration over the range tested of 1.5–14.5 µM (SD = 2.7%; Figure 6b) although variation in BRET^H^ was high (SD = 7.7%) at the lowest bioprobe concentration tested, namely 1.5 µM. The stability of the BRET^H^ ratio across a wide range of measurement conditions is important practically as it indicates the assay can potentially tolerate non-ideal conditions such as variations in flow rate, or reagent concentration or incomplete mixing as long as there is sufficient light for spectrally resolved detection. Although more complete mixing would be predicted to increase the luminosity of the system our data indicate that this would have little effect on the measured BRET^H^ ratio. This potentially simplifies the design requirements for microfluidic devices using BRET^H^ -based detection.

### Comparison of Thrombin Sensitivities between Microfluidic and Static Assay Formats

**Figure 7:**
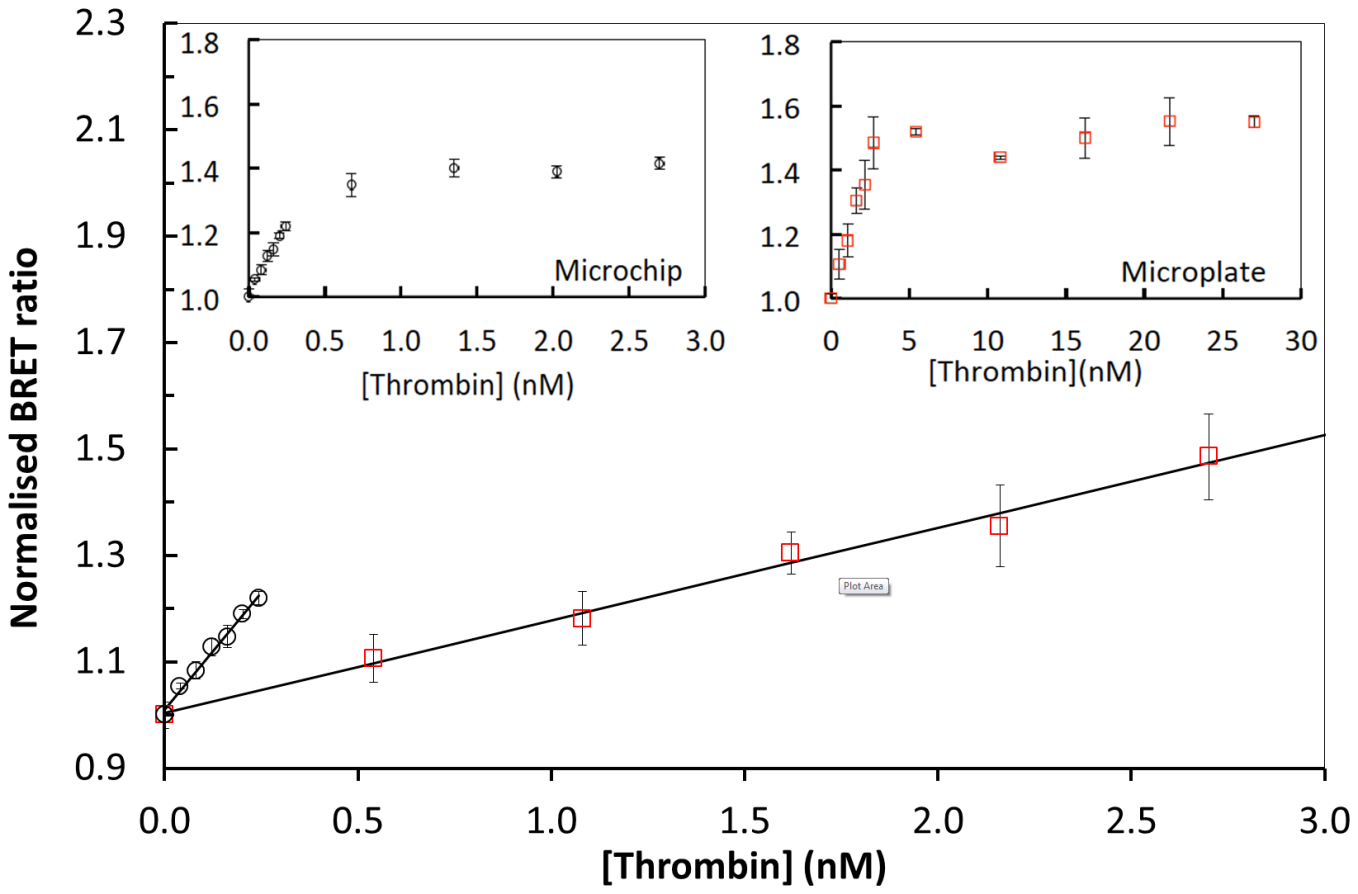
Thrombin sensitivity of the GFP^2^-RG-RLuc bioprobe in microfluidic and multiwall plate formats (mean ±SD, n = 5). For both static and microfluidic assays, BRET^H^ ratios were normalised independently against the ratio measured in the absence of thrombin. All microfluidic measurements were obtained at x = 2.1 mm. (○) microfluidic and (□) multiwell plate measurements. The large graph shows the BRET^H^ responses at low thrombin concentrations. Insets show the corresponding full-range measurements. Data are fitted to linear regressions: y = 0.835x +1.019 (R^2^ = 0.996) for the microfluidic and y = 0.1797x +1.001 (R^2^ = 0.995) for the multiwall.

We compared bioprobe BRET^H^ responses to thrombin on the microfluidic chip and in a standard 96 well microtitre plate format (Figure 7). On the microfluidic chip, the BRET^H^ ratio increased linearly with thrombin concentrations up to 0.24 nM. In the multiwell plate assay, the limit of linearity was 2.7 nM. At thrombin concentrations higher than these, the change in BRET^H^ ratio flattens rapidly, presumably due to digestion of most of the bioprobe before the end of the incubation period. At lower thrombin concentrations, the relationships between BRET^H^ ratio and thrombin concentration were linear, with R^2^ values exceeding 0.995. Comparison of the gradients revealed that the microfluidic method is 4.7 times more sensitive to changing thrombin concentrations than the multiwell plate method. The limit of detection (LOD, see Experimental) for thrombin is 27 pM for the microfluidic chip-based technique compared to 310 pM using the multiwall plate-based technique. The LOD measured in the microfluidic format is intermediate between the values of 15 pM and 53 pM, previously determined for BRET^2^ and BRET^1^, respectively [6], in multiwell plate-based assays. The higher sensitivity observed in the microfluidic system may be due to the smaller detection volume and consequent lack of self-absorption effects as well as the constant refreshing of bioprobe and substrate and removal of potentially inhibitory reaction products. In contrast, in the static multiwell plate assay, unreacted substrate is depleted and potentially inhibitory reaction products accumulate over time. The LOD of 27 pM for the current microchip-based system is much lower than those reported for comparable microfluidic based protease assays. For example, a concentration of 0.001 Unit/mL for proteinase K (i.e. ∼1.2 nM with a specific activity of 30 Units mg^−1^) was used in a microfluidic protease activity assay that used fluorescence polarisation [29]. An LOD of 6.2 nM for trypsin [19] was achieved in an electroluminescence-CCD microchip platform with quantum dot based sensors. The relatively low LOD and high sensitivity of the current technique are extremely attractive and demonstrate the potential usefulness of highly sensitive BRET-based microfluidic sensors for a range of analytes. For example, thrombin concentrations associated with plasma clots have been measured as 0.46 nM using both chromogenic and clotting methods [30]. This is within the range detectable on chip using the Hybrid BRET assay, demonstrating the latter’s potential for directly measuring physiologically relevant concentrations of thrombin.

## Conclusions

Bioluminescence resonance energy transfer has been demonstrated for the first time in a flow format using a fluid phase thrombin-sensitive bioprobe. The BRET^H^ technique used was a hybrid between BRET^1^ and BRET^2^, which allowed testing of the BRET^2^ donor/acceptor components with measurable luminosity. The BRET reaction and detection were carried out in a Y-shape microchannel network in a microfluidic chip. Experiments quantified the effects of measurement location, flow rate and bioprobe concentration. These factors affected the bioluminescence intensities in both optical channels but not the BRET^H^ ratio. The microfluidic technique showed higher sensitivity and lower limits of detection (27 pM) for thrombin compared to the same bioprobe deployed in a multiwell plate-based format and measured with a commercial spectrofluorometer (LOD = 310 pM). The microfluidic biosensor described exemplifies a potentially new class of microfluidics-based BRET sensor devices for chemical and biological sensing and detection.

## Supporting Information

Figure S1 (TIFF)
