# Supplementary figures and images for: Comparison of Static and Microfluidic Protease Assays Using Modified Bioluminescence Resonance Energy Transfer Chemistry
Source: PLoS One. 2014 Feb 14;9(2):e88399. doi: 10.1371/journal.pone.0088399 (PMC3925127; doi:10.1371/journal.pone.0088399)

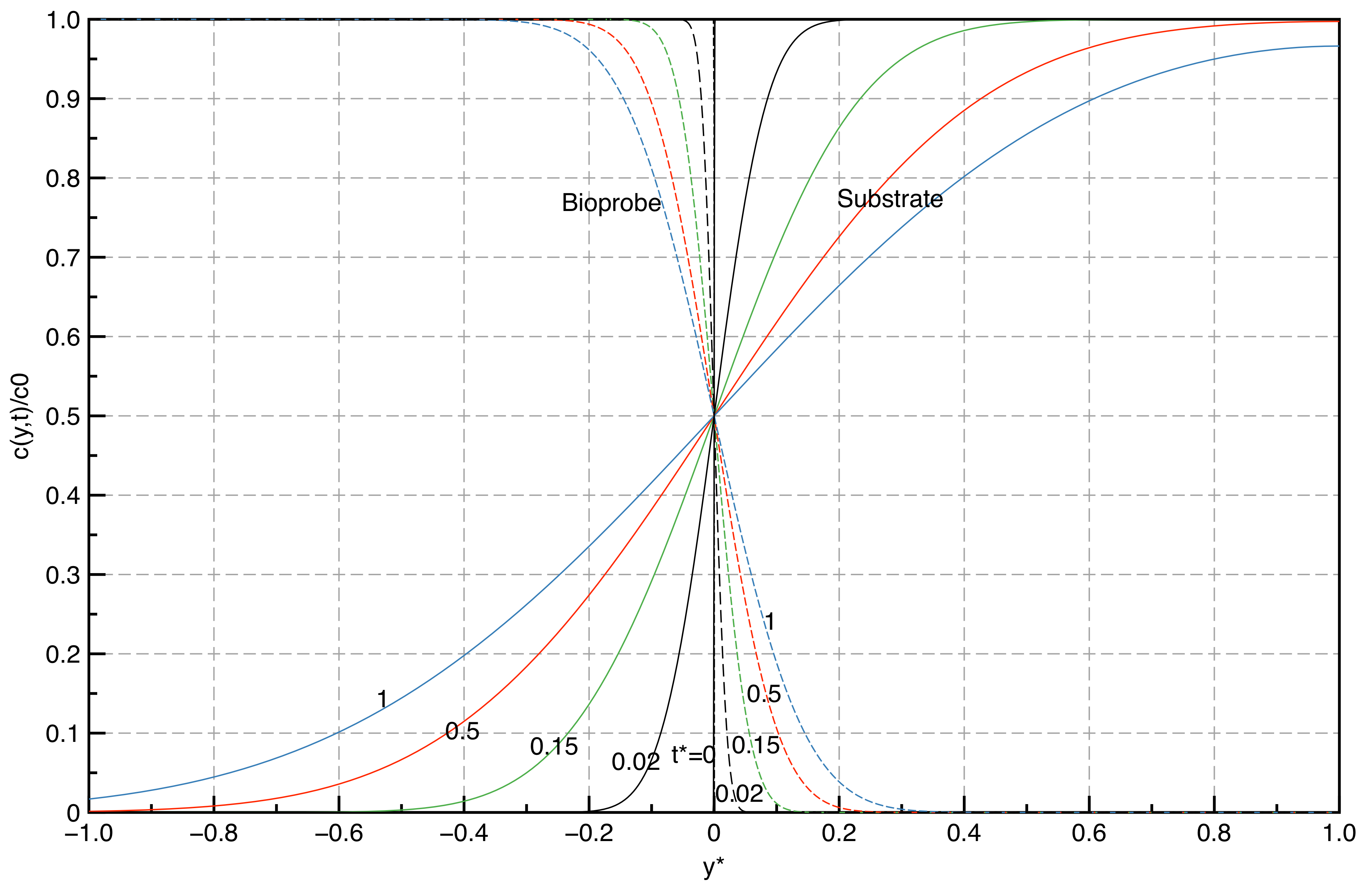

Supplement: Figure S1 — (TIFF) [file pone.0088399.s001.tiff]
